# Axially Loaded Magnetic Resonance Imaging Identification of the Factors Associated with Low Back-Related Leg Pain

**DOI:** 10.3390/jcm10173884

**Published:** 2021-08-29

**Authors:** Tomasz Lorenc, Wojciech Michał Glinkowski, Marek Gołębiowski

**Affiliations:** 1 1st Department of Clinical Radiology, Medical University of Warsaw, 02-004 Warsaw, Poland; tlorenc@wum.edu.pl (T.L.); marek.golebiowski@wum.edu.pl (M.G.); 2 Department of Medical Informatics and Telemedicine, Center of Excellence “TeleOrto” for Telediagnostics and Treatment of Disorders and Injuries of the Locomotor System, Medical University of Warsaw, 00-581 Warsaw, Poland

**Keywords:** low back-related leg pain, low back pain, lumbar spine, magnetic resonance imaging, axial loading, logistic regression

## Abstract

This retrospective observational study was conducted to identify factors associated with low back-related leg pain (LBLP) using axially loaded magnetic resonance imaging (AL-MRI). Ninety patients with low back pain (LBP) underwent AL-MRI of the lumbar spine. A visual analog scale and patient pain drawings were used to evaluate pain intensity and location and determine LBLP cases. The values of AL-MRI findings were analyzed using a logistic regression model with a binary dependent variable equal to one for low back-related leg pain and zero otherwise. Logistic regression results suggested that intervertebral joint effusion (odds ratio (OR) = 4.58; *p* = 0.035), atypical ligamenta flava (OR = 5.77; *p* = 0.003), and edema of the lumbar intervertebral joint (OR = 6.41; *p* = 0.003) were more likely to be present in LBLP patients. Advanced disc degeneration (*p* = 0.009) and synovial cysts (*p* = 0.004) were less frequently observed in LBLP cases. According to the AL-MRI examinations, the odds of having LBLP are more likely if facet effusion, abnormal ligamenta flava, and lumbar facet joint edema are present on imaging than if not. The assessment of lumbar spine morphology in axial loaded MRI adds value to the potential understanding of LBLP, but further longitudinal and loaded–unloaded comparative studies are required to determine the role of acute dynamic changes and instability in LBLP development.

## 1. Introduction

Low back-related leg pain (LBLP) refers to neuropathic pain when the lower back nerves are compromised and is often manifested by sciatic or lumbar radicular pain [1]. LBLP may not be neuropathic and can manifest as a result of non-neuronal structure involvement (e.g., the muscles, ligaments, and disc) in the lumbar spine. Identifying clinically relevant LBLP subgroups is a priority for low back pain (LBP) research. A distinction between different types of LBP is necessary for clinical treatment and research applications, but there is no clear consensus on the definition and identification of LBLP owing to nerve root involvement [2,3]. LBLP is observed to be the more common variant of LBP, occurring in approximately two-thirds of LBP cases. The LBLP subgroup is frequently considered neuropathic when neural tissue in the low back is compromised (e.g., nerve root, dorsal root ganglion), commonly referred to as sciatic or lumbar radicular pain [3,4]. However, LBLP is not always neuropathic.

LBLP can manifest as a result of non-neural structure involvement (e.g., muscle, ligaments, disc) in the lumbar spine (which may likewise affect leg pain). This type of pain is usually referred to as nociceptive pain [3,5]. LBLP coincides with increased disability and health costs compared with LBP [6,7,8], and visitations to primary care units are frequent among LBLP patients [9]. In this group, the prognosis is worse [8], with an increased need for healthcare and more prolonged periods of sick leave from work than low back pain alone [7]. Lumbar spine magnetic resonance imaging (MRI) is widely used to investigate patients with LBP and LBLP, and is a valuable technique to assess disc and facet joint abnormalities [10]; however, it lacks specificity regarding the causes of LBP because these abnormalities are common in asymptomatic subjects [11,12,13]. Variations in symptoms between people with similar recumbent MRI findings are anticipated [14,15,16]. Vertical and horizontal intradiscal pressure measurements are influenced by body position and the degree of disc degeneration according to body position (prone, lateral, upright standing, and upright sitting) [17]. Spinal unloading is effective in reducing intradiscal pressure [18]. Therefore, further exploration of lumbar spine structures must consider the potential generators of LBLP [14]. Findings in patients with position-related differences in pain associated with the size of the intervertebral foramen found on axially loaded MRI (AL-MRI), as opposed to conventional MRI [19,20], are well documented. Changes in forces along the lumbar spine from recumbent to axial loading may, in many cases, result in spinal stenosis, disc herniations, ligamenta flava thickening, and hyperintensity zone appearance, and may result from deformation and displacement of spinal structures that are not observable when recumbent [21]. Recumbent MRI is widely used to investigate patients with LBP and LBLP, but the evidence lacks the prognosis and prognostic factors of LBLP in axially loaded MRI of LBP patients. This evidence could guide timely appropriate diagnosis and referral decisions. We performed an AL-MRI to determine the factors associated with the presence of LBLP.

## 2. Materials and Methods

The study was a single-center, retrospective, observational study conducted in a tertiary hospital. From June 2011 to August 2013, 90 consecutive patients were enrolled in the study. Patients were referred for a lumbar spine MRI with lower back pain as an indication. The Institutional Bioethical Review Board approved this study and written informed consent was obtained from all participants. Adult patients aged 21 to 89 were enrolled in this study after orthopedic consultation and clinical evaluation to exclude hip or knee-related pain. Patients completed self-reported questionnaires, and the referring physicians performed the clinical assessment. No strict neurological examinations were performed at the time of the MRI. A visual analog scale (VAS) and patient pain drawings were obtained directly before the MRI [22,23].

Patients were examined consecutively following the National Health Fund waiting list for diagnostic imaging examinations and following the diagnostic workflow with no priorities owing to the research protocol. Exclusion criteria included significant spinal deformity or fracture, osteoporosis, previous spine surgery, lack of patient compliance, a body mass below 40 kg, and a lack of written consent from the patient. General contraindications for MRI examinations (e.g., pacemakers, ferromagnetic implants, foreign bodies, and claustrophobia) were also considered.

### 2.1. Axially Loaded MRI

The examination was performed using a 1.5 T MRI (Ingenia, Philips Healthcare, Eindhoven, the Netherlands). Axial loaded MRI was applied using an external nonmagnetic DynaWell (DynaWell L-Spine, DynaWell Diagnostics, Las Vegas, NV, USA) compression device, and examinations were performed with a 3D T2-weighted volume isotropic turbo spin-echo acquisition (VISTA) sequence (Table 1). According to previous disc pressure measurements [24], the chosen load equaled 40–50% of the patients’ body weight, with equal load distribution on both legs (20–25% of body mass per leg). The patient was subjected to this load in the lying position for at least 5 min before the examination. Images in the recumbent position were acquired using a standard protocol (Table 1).

### 2.2. Image Analysis

**Figure 1:**
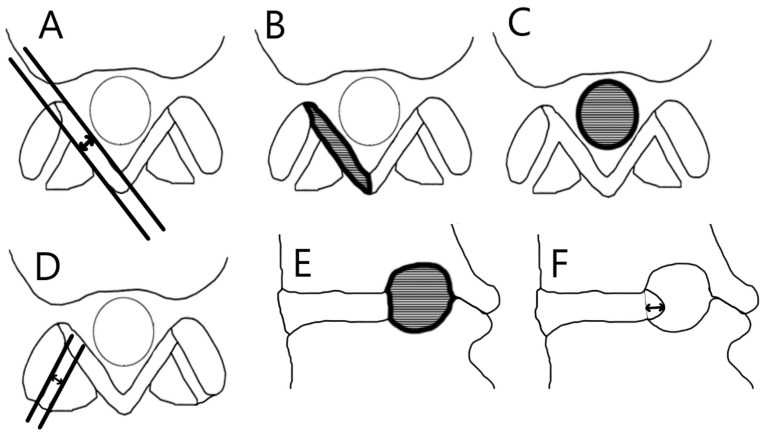
The image analysis. (**A**) Thickness of the ligamentum flavum; (**B**) cross-sectional area of the ligamentum flavum; (**C**) the dural sac cross-sectional area; (**D**) thickness of facet joint effusion; (**E**) the sagittal cross-section area of the vertebral foramen; (**F**) sagittal diameter of the herniated disc.

Images were assessed on a dedicated workstation (IntelliSpace Portal, Philips Healthcare, Eindhoven, The Netherlands) at a single center. Disc herniation was assessed on axial-loaded images on all levels according to the Michigan State University (MSU) classification [25]. The size of the herniated disc was determined by measuring the largest sagittal diameter on each disc level (if present) on the images (Figure 1). The dural sac cross-sectional area was calculated for each level from L1-L2 to L5-S1 to examine axial loading. Measurements were performed by encircling the area of the dural sac transversely, parallel to the vertebral endplates, capturing T2-weighted MRI images with the plane precisely positioned at the midplane of the intervertebral disc (Figure 1). The degree of spinal stenosis was assessed at all levels according to the classifications of Schizas et al. [26]. The vertebral foramina sagittal cross-section area was determined for each level, from L1-L2 to L5-S1, on both sides. Measurements were performed by encircling the vertebral foramina area in sagittal cross sections for the phase with axial loading (Figure 1). The degree of foraminal nerve compression was assessed at all disc levels on both sides using the system introduced by Lee et al. [27]. The thickness and cross-sectional area of the ligamentum flavum were determined for L1-L2 to L5-S1. Thickness was measured at the middle of the ligamentum flavum, and area measurements were captured by encircling the cross-section area of the ligamentum flavum at the facet joint level (Figure 1). Synovial cyst presence and size were determined by measuring the largest cyst area (mm^2^). The presence and thickness (mm) of facet joint effusion were also assessed (Figure 1). The degree of disc and facet joint degeneration was assessed on recumbent images at all disc levels according to the classifications of Pfirrmann et al. [28] and Weishaupt et al. [29], respectively.

### 2.3. Clinical Evaluation

A double clinical evaluation was performed. During the orthopedic consultation, the first referred the patient for MRI before putting the patient on the waiting list for the diagnostic imaging and face-to-face, right before examination by the researcher (principal investigator) using interview and survey forms. Pain intensities were evaluated separately for low back and legs using a VAS [22]. Pain location was determined using patient pain drawings: an outline of a human figure on which the patient marks the areas where they experience pain [23]. Patients were asked to complete the drawings, which were then scored for presence or absence of pain in 45 body areas. We defined LBLP as pain radiating to at least one leg area on the pain drawing, and a leg VAS scale intensity of ≥6. Areas of the buttock and the front side of the thigh were not included as LBLP.

The evaluation of symptoms accompanying potential spinal stenosis was performed using the Zurich Claudication Questionnaire (ZCQ), with two distinct domains that involve symptom severity and physical function [30]. The ZCQ is a disease-specific self-report outcome instrument commonly used in spine-related disability scoring, particularly for patients with lumbar spinal stenosis (LSS), and quantifies the severity of symptoms, physical function characteristics, and patient satisfaction. It was designed to complement existing generic measures of lumbar spine disability and health status in evaluating patients with LSS. Seven questions in the symptom severity domain focus on the frequency and severity of back, buttock, and leg pain and range from 1 (none) to 5 (very severe). The five questions in the physical function domain focus exclusively on a patient’s ability to walk, and range from 1 (comfortably) to 4 (none); the first question asks the patient to list a distance range that he or she can walk. The score represents a percentage of the maximum possible score. The result increases with worsening disability.

Self-reported disability related to LBP was measured using the Oswestry Disability Index (ODI, Version 2.1a), polish translation [31] of the originally developed questionnaire by Fairbank et al. [32,33], a commonly used outcome measure for patients with low back pain. The psychometric properties of the ODI determine the questionnaire’s suitability as a useful clinical tool. The ODI score describes the degree of disability related to LBP. Scores from 0% to 20% indicate minimal disability; 20% to 40%, moderate disability; 40% to 60%, severe disability; 60% to 80%, crippled; and 80% to 100%, bedbound or exaggerating [32]. The Beck Depression Inventory (BDI) was used to assess the presence and severity of depression [34], and is a 21-item, self-report rating inventory that measures characteristic attitudes and symptoms of depression. Total score BDI ranging from 0 to 13 is considered minimal; 14 to 19, mild; 20 to 28, moderate; and 29 to 63, severe.

### 2.4. Statistical Analysis

Logistic regression modeling was used to identify independent MRI findings associated with LBLP. A binary dependent variable was defined, with one for LBLP and zero for the other. We assumed that LBLP occurred if the leg VAS scale intensity was ≥6, and pain radiated to at least one area of the leg on the pain drawing (areas for the buttocks and the front side of the thigh were not included for LBLP).

The following variables to potentially predict the respective outcome in LBLP were included in the model:

- Dural sac cross-sectional area with axial loading;
- Grade of lumbar spinal canal stenosis with axial loading;
- Disc herniation according to MSU with axial loading;
- Intervertebral disc herniation size with axial loading;
- Hyperintensity zone size with axial loading;
- Type of ligamenta flava with axial loading;
- Cross-sectional area of the intervertebral foramen with axial loading;
- Foraminal stenosis, according to Lee et al. [27] classification with axial loading;
- Intervertebral disc degeneration, according to Pfirrmann et al. [28] classification;
- Facet joint degeneration, according to Weishaupt et al. [29] classification;
- Lumbar facet joint edema;
- Synovial cyst area with axial loading;
- Facet effusion with axial loading.

Qualitative variables were categorized based on published classifications. Continuous variables were categorized based on cut-off values, either from the literature, clinical indications, or statistical reasons. The results of the Schönström et al. [35,36] studies indicated that constriction of the cauda equina to a size less than ~105 mm^2^ leads to the pressure increase. Therefore we categorized the dural sac cross-sectional area into <105, 105–145, and ≥145 mm^2^. According to Schizas et al. [26], lumbar spinal canal stenosis was graded as A1 (no spinal canal stenosis) and ≥A2. Lumbar disc herniation was graded according to the MSU [25] classification as 0 (no lumbar disc herniation) and ≥1a. Disc herniation size was divided into equinumerous groups to maximize statistical test power (< 3; 3–5; ≥5 mm). Hyperintensity zone, lumbar facet joint edema, synovial cyst, and facet effusion were divided into two groups: absent (0) or present (>0). Ligamenta flava were divided into typical (thin with small cross-sectional area (thickness < 5 mm; area < 55 mm^2^) or thick with large cross-sectional area (thickness ≥ 5 mm; area ≥ 55 mm^2^)) and atypical (thin with large cross-sectional area (thickness < 5 mm; area ≥ 55 mm^2^) or thick with small cross-sectional area (thickness ≥ 5 mm; area < 55 mm^2^)). The intervertebral foramen area was divided into equinumerous groups to maximize statistical test power (<93; 93–117; ≥117 mm^2^). Foraminal stenosis was graded according to the Lee et al. [27] classification as 0 (no foraminal stenosis) and >0. Intervertebral disc degeneration was graded according to the Pfirrmann et al. [28] classification as <4 (intermediate gray signal intensity, normal disc height) and ≥4. Facet joint degeneration was graded according to the Weishaupt et al. [29] classification as ≤1 (normal or mild degenerative disease) and >1. The modeling process was performed using the stepwise backward selection method with inclusion and exclusion criteria of 0.1 and 0.05, respectively.

Comparison of chi-square tests of the characteristics’ distributions (categorical variables) or the Mann–Whitney U test (continuous variables) were used. Statistical analysis was performed using IBM SPSS Statistics (IBM Corp., Armonk, NY, USA) version 23 for Linux.

## 3. Results

Baseline characteristics of the study population are presented in Table 2.

No significant difference between the LBLP and no-LBLP groups was found for demographics characteristics (Table 3).

The categories and frequencies of radiological variables included in the logistical regression model are provided in Table 4.

The results indicate that the odds of having LBLP are 4.58, 5.77, and 6.41 times more likely if facet effusion, atypical ligamenta flava, and lumbar facet joint edema are present, respectively, on AL-MRI imaging than if they are not. Conversely, according to the AL- MRI examinations, the odds of having LBLP are 0.138 and 0.165 times less likely if synovial cyst and advanced intervertebral disc degenerations are present, respectively, on imaging than if they are not. The final logistic regression model parameters are presented in Table 5.

## 4. Discussion

Owing to significant differences in terms of subjective symptoms expressed by worse results, particularly low back VAS, ODI, and ZCQ in the LBLP group, with similar demographic characteristics in both groups, examining the factors observed in the AL-MRI study was considered highly desirable. This observational study demonstrated a relationship between axially loaded radiological findings and LBLP. The lumbar facet joint edema, facet effusion, and atypical ligamenta flava are independently associated with the presence of LBLP. Compared with previous studies concerning the association between AL-MRI and LBLP, this is somewhat complicated owing to the availability of only recumbent MRI studies. Previous recumbent MRI studies evaluated a small group of radiological variables (dural sac and disc dimensions) [37] or were limited to one imaging variable (nerve root compression) [6,38,39]. In an ATLAS study [6,39], MRI findings of nerve root compression were correlated with patient clinical presentations; however, the authors included the study routine MRI from the primary care setting. In LBLP cases, the anteroposterior diameter of the intervertebral disc prolapse and the dural sac was measured by Pneumaticos et al. [37]. A threshold value of 10 mm for the dural sac AP diameter was sensitive and specific for LBLP in a small patient population. This investigation provided evidence that more than 3 mm of disc herniation was sensitive and specific for LBLP; however, if the AP diameters of herniated discs in symptomatic patients were compared with similar measurements in asymptomatic controls, the most sensitive and specific threshold value was 6.8 mm. Pneumaticos et al. observed a significant overlap in disc herniation size in symptomatic and asymptomatic individuals and found it to be a source of potential limitation of this measurement [37]. The investigation did not demonstrate that disc herniation size and dural sac cross-sectional area were significant predictors of the occurrence of LBLP in a group of LBP patients.

**Figure 2:**
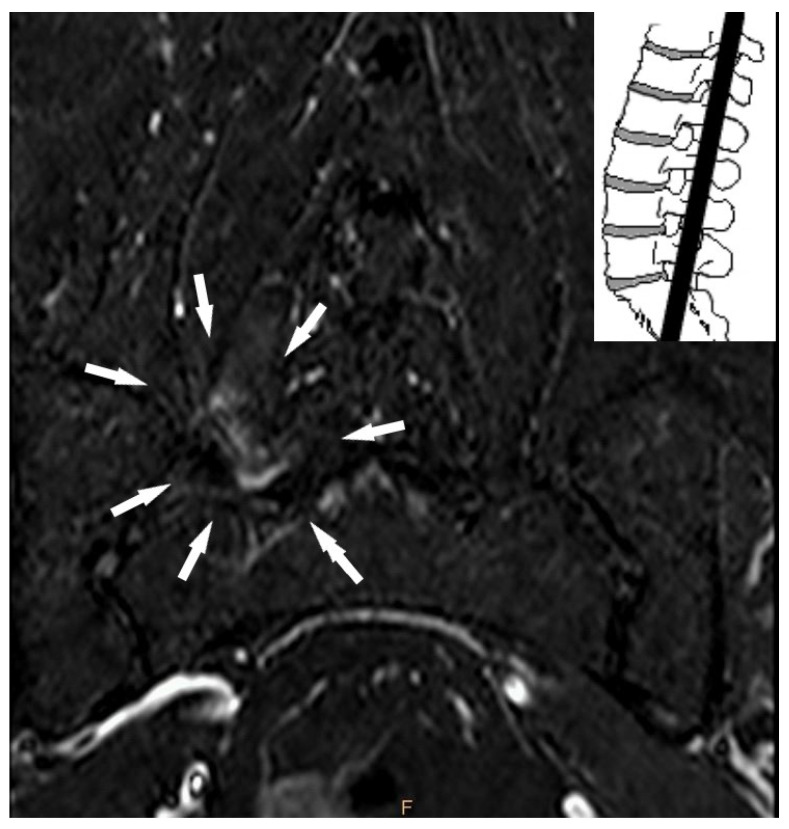
Axial short tau inversion recovery (STIR) T2-weighted sequence showing right L5-S1 facet joint bone marrow edema signal (white arrows).

The present study also evaluated other factors associated with the presence of LBLP in AL-MRI, which have not been previously covered in the literature. In our study population, facet joint edema was found in a considerable percentage of patients with LBP (41.1%) and was mainly found at the L4-L5 and L5-S1 levels (Figure 2). So-called bone marrow edema is a common cause of acute pain in the musculoskeletal system and is believed to predict LBP originating from posterior components, such as the facet joints [12,40,41]. The pathogenesis of facet joint edema is unclear, but, like the peripheral joints, overload and degenerative changes may be associated with signal abnormalities [19]. Joint overload may occur in malalignment, neuromuscular dysfunction, ligament loosening, and repetitive trauma. The symptoms of facet joint osteoarthritis can mimic those associated with disk herniation through the so-called “pseudo-radicular” referral pattern, which often makes it difficult to distinguish between the two conditions [12]. According to the AL-MRI examinations, lumbar spine posterior element abnormalities were frequently associated with LBLP.

**Figure 3:**
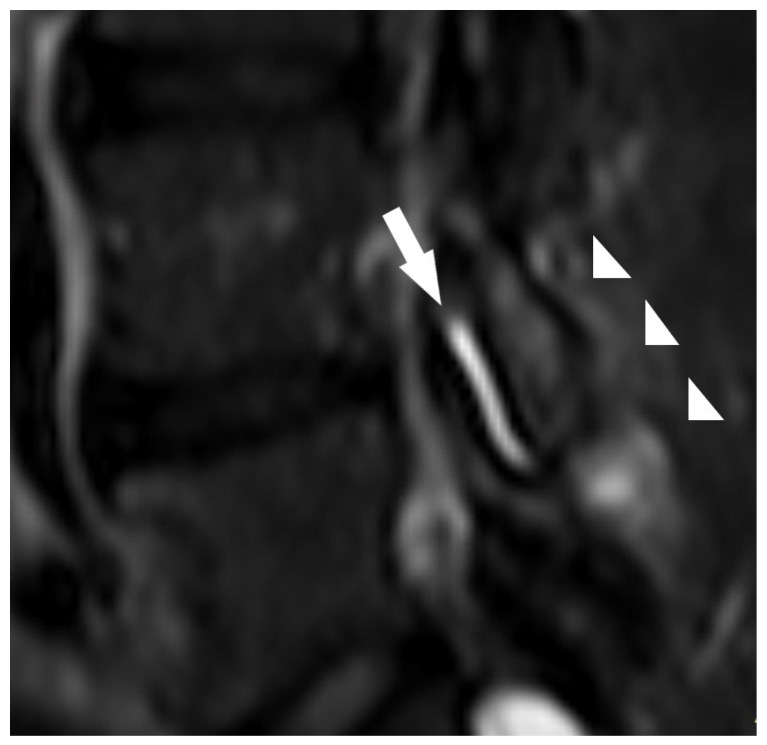
Magnetic resonance image showing the LBLP case with facet effusion (arrow), bone marrow, and surrounding soft-tissue edema (arrowheads).

The prevalence of facet effusion in the lumbar spine was 20.0% and was most observed at the L4-L5 and L5-S1 levels (Figure 3). Facet effusion represents an MRI finding describing the accumulation of fluid in the facet joint. Facet effusion may represent a cause of LBP or a manifestation of the cause of LBP originating from posterior components such as the facet joints [42,43], but according to some reports, this association is controversial [44]. Moreover, several studies have suggested that fluid collection detected within the lumbar facet on MRI indicates lumbar spine segmental instability, defined as hypermobility (especially translation between two contiguous vertebrae) or abnormal mobility [45,46,47]. They concluded that facet joint effusion on MRI should increase suspicion of lumbar spine instability.

The possibility of a relationship between the ligamentum flavum and lumbar spine instability has been reported [48]. Kirkaldy-Willis et al. [49] described three stages in the natural history of disc degeneration, whereby the second stage is associated with hypermobility and degeneration of the facet joints, resulting in capsular and ligamenta flava laxity and instability. Ligamenta flava that are thin with a large cross-sectional area could be more vulnerable to instability and could be associated with mechanical insufficiency at early stages. On the other hand, thick ligamenta flava with a large cross-sectional area are recognized as a final stage of self-limiting treatment of chronic lumbar instability [50]. Degenerative changes secondary to the aging process and mechanical stress due to chronic spinal instability are two pathomechanisms that have been proposed for the development of ligamenta flava thickening [51]. Nevertheless, further research should be conducted to explain LBLP beyond simple nerve compression by hypertrophied ligamenta flava.

Interestingly, advanced intervertebral disc degeneration showed a reverse association with LBLP. The odds of having LBLP are about six times less likely if advanced intervertebral disc degenerations are present on imaging than if they are not. These findings could indicate a less critical role of advanced disc degeneration and disc-related cause in LBLP development. The explanation for the reverse association between a synovial cyst and LBLP is somewhat difficult. Intuitively, one would expect that LBLP would be strongly linked to a synovial cyst and nerve compression. For example, Kouyialis et al. [52] observed increased synovial cysts as causative agents of LBLP. Bydon et al. [53] reported recurrent back pain occurring in a significant number of patients who underwent synovial cyst surgical decompression. A synovial cyst may reflect progressive degenerative changes of the ligamentum flavum. Progressive degeneration would be consistent with the low prevalence of LBLP associated with advanced disc degeneration—a low prevalence of LBLP observed in patients with advanced degenerative changes and chronic symptoms.

In summary, atypical ligamenta flava, facet joint edema, and facet effusion can reflect spinal segmental instability. Therefore, it was hypothesized that the spine’s posterior elements and segmental instability might be more frequently associated with LBLP than direct nerve compression (advanced intervertebral disc degeneration and/or synovial cysts). Moreover, facet joint edema and facet effusion can also reflect acute changes in the spine, in contrast to advanced intervertebral disc degeneration and synovial cysts, representing chronic changes. Therefore, we also hypothesized that acute spine changes might be more frequently associated with LBLP than chronic changes.

Our study has some limitations. The static MRI results with positive findings for spinal pathologies are known from rare publications in some asymptomatic individuals [16]. LBLP symptomatic patients with no MRI findings were also described in the literature [54]. However, enrollment in this study consisted of LBP and LBLP symptomatic patients. The retrospective approach may deliver a possibility of bias. Some radiological variables were merged to reduce the number of categories in the model to achieve sufficient power in multivariate logistic regression analysis.

Another limitation may derive from the time gap between referral physician examination and the date of the MRI imaging, owing to no strict neurological examinations being performed at the time of MRI. The symptoms could change over even a relatively short time. However, VAS and pain mapping have relatively good reliability and validity with neurological examinations concerning pain occurrence and localization [22,23].

We assume that, considering perfectly aimed minimally invasive endoscopic or navigated surgery, axially loaded imaging is needed. Precision in targeting the pain generator during endoscopic spinal surgery intervention can elicit more perfectly aimed procedures, with lower surgical risk. AL-MRI of the lumbar spine should be considered an additional procedure on patients with LBLP. Further research is needed to document the improvements and convince clinicians, researchers, stakeholders, and commissioners that this type of MRI examination should be considered routine.

## 5. Conclusions

Logistic regression used in this study determined which spine components were significantly associated with the presence of LBLP. Facet effusion, lumbar facet joint edema, and atypical ligamentum flavum are identified as variables independently associated with LBLP. According to the AL-MRI examinations, the odds of having LBLP are 4.58–6.41 times more likely if facet effusion and lumbar facet joint edema are present on imaging than if they are not. Moreover, the odds of having LBLP are 5.77 times more likely if abnormal ligamenta flava are observed with AL-MRI examinations than if they are not. The logistic regression results suggest that the presence of advanced intervertebral disc degeneration (*p* = 0.009) and synovial cysts (*p* = 0.004) indicate a decreased occurrence of LBLP. Assessment of lumbar spine morphology in AL-MRI adds value to the potential understanding of LBLP, but further longitudinal and loaded–unloaded comparative studies are required to determine the role of acute dynamic changes and instability in low back-related leg pain development.

## Figures and Tables

**Table 1:** High-resolution 3D VISTA magnetic resonance pulse parameters and a standard protocol for lumbar spine MRI.

Parameters	3D VISTA T2	STIR Sagittal	2D TSE T2 Sagittal	2D TSE T2 Axial	2D TSE T1 Sagittal
TR/TE, ms	2000/90	2000/70	2000/120	3000/120	400/13
NSA	1	2	3	3	2
FOV, mm	300 × 200	300 × 160	300 × 160	300 × 160	300 × 160
Acquisition matrix	300 × 196	132 × 198	200 × 300	332 × 223	180 × 238
Acquisition voxel, mm	1.0 × 1.0 × 0.5	1.2 × 1.5 × 4.0	0.9 × 1.0 × 4.0	0.6 × 0.8 × 3.0	0.9 × 1.3 × 4.0
Reconstruction matrix	640	432	640	400	480
Reconstruction voxel, mm	0.47 × 0.47 × 0.5	0.7 × 0.7 × 4.0	0.47 × 0.47 × 4.0	0.5 × 0.5 × 3.0	0.6 × 0.6 × 4.0
Turbo factor	61	33	42	24	7
Scan time, min	06:46	03:33	03:44	04:12	02:38

TR, repetition time; TE, echo time; NSA, number of signal averaging; FOV, field of view; VISTA, volume isotropic turbo spin-echo acquisition; STIR, short tau inversion recovery; TSE, turbo spin-echo; MRI, magnetic resonance imaging.

**Table 2:** Baseline characteristics of patients included in the study.

	Number, *n*	Percentage, %
Outcome		
LBLP patients	38	42.2
No-LBLP patients	52	57.8
Age categories		
≤40 years	32	35.6
41–60 years	35	38.9
>60 years	23	25.6
Sex		
Male	46	51.1
Female	44	48.9
BMI categories		
18 ≤ BMI < 25 kg/m^2^	44	48.9
25 ≤ BMI < 30 kg/m^2^	27	30.0
≥30 kg/m^2^	19	21.1
ODI		
≤40	59	65.6
>40	31	34.4
ZCQ		
<40	23	25.6
40–60	49	54.4
>60	18	20.0
BDI		
<14	69	76.7
≥14	21	23.3

LBLP, low back-related leg pain; BMI, body mass index; VAS, visual analog scale; ODI, Oswestry Disability Index; ZCQ, Zurich Claudication Questionnaire; BDI, Beck Depression Inventory.

**Table 3:** Comparison of patients with (LBLP) and without (no-LBLP) low back-related leg pain.

	LBLP (*n* = 38)	No-LBLP (*n* = 52)	*p*
Age, yrs	53	46	0.393
Male, *n*	19 (41.3)	27 (58.7)	0.513
BMI, kg/m^2^	26	25.5	0.15
BDI	8.5	5.5	0.117
Low back VAS	6	5	0.002 *
ODI	43	26	<0.001 *
ZCQ	34.5	27	<0.001 *

Note: All figures are medians unless stated otherwise as frequencies. Unless otherwise stated, percentages are in parentheses. * indicates *p* < 0.05. BMI, body mass index; BDI, Beck Depression Inventory; VAS, visual analog scale; ODI, Oswestry Disability Index; ZCQ, Zurich Claudication Questionnaire.

**Table 4:** Categories and frequencies of variables obtained by axially loaded MRI included in the logistical regression model.

Variables	Categories	Number with Feature, *n*	Rate, %
Dural sac cross-sectional area, mm^2^	<105	29	32.2
	105–145	29	32.2
	≥145	32	35.6
Grade of lumbar spinal canal stenosis according to Schizas et al. [26] classification	A1	44	48.9
	≥A2	46	51.1
Disc herniation according to the MSU [25] classification of lumbar disc herniation	0	28	31.9
	≥1a	62	68.9
Disc herniation size, mm	<3	32	35.6
	3–5	22	24.4
	≥5	36	40.0
Hyperintensity zone size, mm	0	61	67.8
	>0	29	32.2
Ligamentum flavum type (typical, atypical)	Typical (thickness < 5 mm and area < 55 mm^2^)	24	26.7
	Typical (thickness ≥ 5 mm and area ≥ 55 mm^2^)	26	28.9
	Atypical (thickness < 5 mm and area ≥ 55 mm^2^)	38	42.2
	Atypical (thickness ≥ 5 mm and area < 55 mm^2^)	2	0.2
Intervertebral foramen area, mm^2^	<93	30	33.3
	93–117	30	33.3
	≥117	30	33.3
Foraminal stenosis, according to Lee et al. classification [27]	0	35	27.8
	>0	65	72.2
Intervertebral disc degeneration, according to Pfirrmann et al. classification [28]	<4	17	18.9
	≥4	73	81.1
Facet joint degeneration, according to Weishaupt et al. classification [29]	≤1	42	46.7
	>1	48	53.3
Lumbar facet joint edema	Absent	53	58.9
	Present	37	41.1
Synovial cyst area, mm^2^	0	62	68.9
	>0	28	31.1
Facet effusion thickness, mm	0	72	80.0
	>0	18	20.0

MSU, Michigan State University.

**Table 5:** Radiologic factors associated with LBLP.

Variables	B	SE	*p*-Value	OR	95% CI	
					Upper	Lower
Intervertebral disc degeneration according to Pfirrmann classification ≥ 4	−1.804	0.690	0.009	0.165	0.043	0.637
Facet effusion thickness, mm > 0	1.522	0.721	0.035	4.580	1.114	18.830
Lumbar facet joint edema (present)	1.858	0.617	0.003	6.412	1.913	21.492
Synovial cyst area, mm^2^ > 0	−1.984	0.692	0.004	0.138	0.035	0.534
Atypical ligamenta flava (thickness < 5 mm; area ≥ 55 mm^2^)	1.753	0.593	0.003	5.771	1.804	18.461
Constant	−0.170	0.583	0.771	0.844		

B, beta; SE, standard error; OR, odds ratio; CI, confidence interval.

## Data Availability

The dataset analyzed is not publicly available, but is available from the corresponding author on reasonable request.
